# Novel banana-discotic hybrid architectures

**DOI:** 10.3762/bjoc.5.52

**Published:** 2009-10-07

**Authors:** Hari Krishna Bisoyi, H T Srinivasa, Sandeep Kumar

**Affiliations:** 1 Raman Research Institute, C. V. Raman Avenue, Sadashivanagar, Bangalore, India. Phone: +91 80 23610122, Fax: +91 80 23610492

**Keywords:** bent-core, dimer, discotic, triphenylene

## Abstract

Here we present the design and synthesis of novel banana-discotic dimers and banana-bridged discotic dimers. The chemical structures have been characterized by spectral techniques and elemental analysis. The thermal behaviors of the compounds have been investigated by polarizing optical microscopy and differential scanning calorimetry. None of these synthesized compounds exhibit any liquid crystalline property probably because of the incompatibility of the bent-core with the discotic core.

## Introduction

Liquid crystals are unique functional self-organized soft materials which possess order and dynamics. Recently, banana liquid crystals or bent-shaped mesogens have attracted considerable research interest in the field of soft condensed matter. The mesomorphic properties of a variety of bent shaped molecules have been investigated quite extensively. The polar order of these molecules, due to their bent shape, display interesting properties such as ferroelectric or anti-ferroelectric switching [1–7]. The occurrence of superstructural chirality in the mesophase of bent-core compounds with no inherent chirality is not only of fundamental scientific interest but also of industrial application as this chirality can be switched in external electric fields. Various new applications of these materials include nonlinear optics, flexoelectricity, photoconductivity, molecular electronics and the design of biaxial nematic phases [8–9]. In addition to various banana phases, these mesogens also display classical nematic, smectic and columnar phases.

On the other hand, the unique geometry of the columnar mesophase formed by discotic liquid crystals is of great importance not only as models for the study of one-dimensional charge and energy migration in organized systems but also as functional materials for device applications such as one-dimensional conductors, photoconductors, light emitting diodes, photovoltaic solar cells, gas sensors etc. [10–21]. The functional capabilities of these materials are due to their easier processibility, spontaneous alignment between electrodes and self-healing of defects owing to their dynamic nature. Furthermore, there has been considerable interest in the field of non-conventional low molar mass liquid crystals, especially in liquid crystal dimers because of their interesting mesomorphic properties. Liquid crystal oligomers serve as ideal models for polymers and networks due to the striking similarity in their transitional behavior and like polymers, some oligomers form glassy mesophases [22–26]. Their purification and characterization are easy unlike polydisperse polymers. Owing to the restricted motion of the components, liquid crystal oligomers provide and stabilize a variety of fluid phases with fascinating functions and oligomeric approach provides a wide flexibility in the molecular design.

The hybridization of above mentioned two varieties of liquid crystals may lead to novel nanostructured materials with interesting physical properties important for device applications. If such architectures could provide columnar mesophases then the charge carrier mobilities are expected to increase owing to the presence of conducting aromatic bent-cores in the otherwise insulating alkyl chain mantle around the discotic cores. Hence these compounds are anticipated to display superior electronic and optoelectronic performance in organic semiconducting devices. Moreover, these hybrids may also display new types of banana phases whose potential applications have been mentioned in the introductory paragraph. With this idea in mind, we have designed and synthesized novel banana-discotic dimers. A typical five-ring banana liquid crystal and triphenylene based discotic [27] liquid crystals were chosen to prepare these novel dimers. Here we report the synthesis, characterization and phase behavior of these novel dimers.

## Results and Discussion

The bend in the rigid cores of the banana liquid crystal compounds leads to a reduction of the rotational disorder of the molecules around their long axes. If segregation of aromatic cores and aliphatic chains is sufficiently strong, the molecular structure facilitates an organization into layers. Since the molecules are closely packed within the smectic layers and additionally, the rotation about their long axes is strongly hindered, the bent directions align parallel in each layer. As a result of this directed organization, each layer develops a spontaneous polarization. On the other hand, most of the discotic liquid crystalline compounds form columnar phases because of the strong π-π interaction of poly aromatic cores. In the columnar mesophases, the discotic molecules stack one on top of the other to form columns and the columns so formed arrange themselves in various two dimensional lattices.

**Scheme 1:**
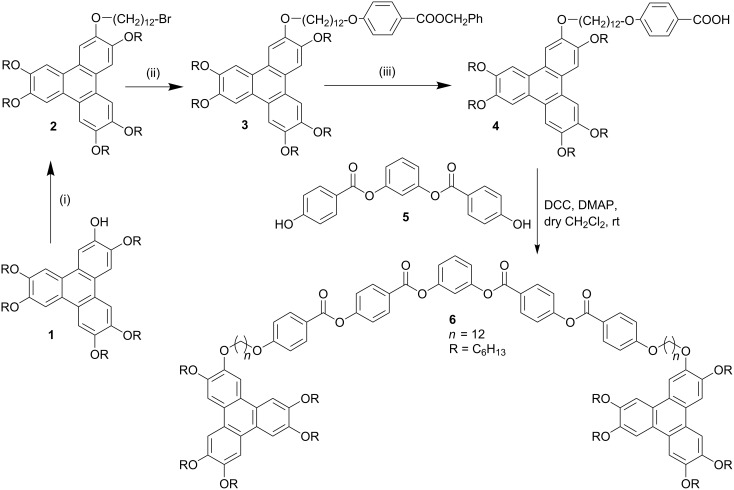
Synthesis of banana bridged discotic dimer. Reagents and conditions; (i) Br(CH_2_)_12_Br, Cs_2_CO_3_, MEK, 85 °C; (ii) HO–C_6_H_4_–COOCH_2_Ph, Cs_2_CO_3_, MEK, 85 °C; (iii) 5% Pd-C, H_2_, 1,4-Dioxane, r.t.

**Scheme 2:**
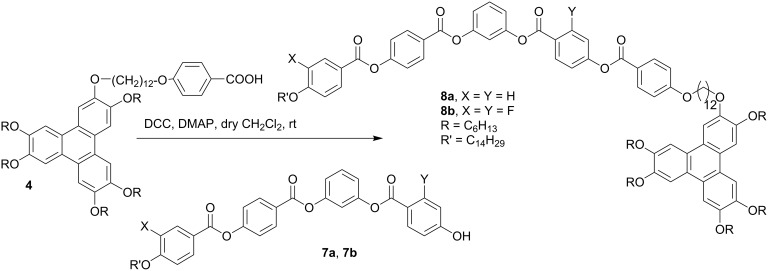
Synthesis of banana-discotic dimers.

The synthesis of the target compounds, banana-bridged discotic dimer **6** and banana-discotic dimers **8a** and **8b** are shown in Scheme 1 and Scheme 2, respectively. In compound **6** a five-ring banana unit joins two triphenylene discotic cores via two flexible alkyl spacers whereas in compound **8a** and **8b** a five-ring banana unit joins to a triphenylene core via a flexible alkyl spacer.

The chemical structures of the final compounds have been characterized by spectral techniques and elemental analysis. The analytical data is in good agreement with their chemical structures. The phase behavior of the novel compounds have been observed by polarizing optical microscopy and differential scanning calorimetry. All the solvent crystallized compounds pass from crystalline state to isotropic liquid state on heating, and on cooling they crystallize into the solid state without any intervening mesophase. The transition temperatures and their associated enthalpies of the novel compounds are listed in Table 1.

**Table 1:** Transition temperatures (°C, peak temperature) and enthalpies (kJ/mol in parentheses) of the banana-discotic dimers both on heating and cooling at the scan rate of 5 °C/min.

Compound	Heating	Cooling

**6**	Cr^a^ 51.1 (38.8) I^b^	I 38.7 (15.4) Cr
**8a**	Cr 92.6 (57.7) I	I 77.6 (43.6) Cr
**8b**	Cr 75.8 (40.9) I	I 54.9 (35.2) Cr

^a^Cr = Crystalline solid, ^b^I = Isotropic liquid

The symmetric dimer **6** melts at 51.1 °C on heating and crystallizes at 38.7 °C on cooling. Under polarizing optical microscopy we do not see any other transition, which was confirmed by differential scanning calorimetry measurements. The absence of either B-phase or discotic columnar mesophase in compound **6** may be explained as follows: since the five-ring banana unit, which otherwise exhibits mesophase with two terminal alkyl chains, is now attached with two bulky triphenylene units at the ends, the packing of the bent-cores in layers will not be favored. In other words, the bent core-bent core interactions are too weak to produce any B-phase. On the other hand, the two triphenylene units in compound **6** when attached with flexible methylene spacers exhibit columnar mesomorphism. However, in compound **6** they are attached to each other with a intervening rigid bent core unit which is probably not allowing the two discs to lie in the same plane and hence hinders columnar mesomorphism in this architecture. So non-coplanarity of the discs and the rigid bent-core unit in the spacer seems to be the reason for absence of liquid crystallinity in compound **6**. The banana discotic hybrid **8a** melts at 92.6 °C to an isotropic liquid and on cooling it slowly crystallizes at 77.6 °C. Similarly, compound **8b** on heating melts to isotropic liquid at 75.8 °C and on cooling slowly crystallizes at 54.9 °C. These polarizing optical microscopy observations are confirmed by differential scanning calorimetry measurements. The absence of mesomorphism in this architecture may arise from two competing factors. First, the bent core units will try to pack in layers by microsegregation but in this kind of packing arrangement the attached discs have to lie side by side which is not a preferable option for discs. Moreover, the cross-sectional area of the disc is such that it will not allow efficient packing of the bent cores in layers. Secondly, when the discs try to stack one above the other to produce columnar phases, the bent core unit will disrupt it owing to its rigidity and high volume fraction compared to the other alkyl chains surrounding the triphenylene disc which otherwise provide space filling effect for columnar mesomorphism. The difluoro compound **8b**, has a lower melting point than its hydrocarbon parent compound **8a** which is very common observation in bent-core compounds.

## Conclusion

We have designed and synthesized banana-discotic dimers and a banana-bridged discotic dimer to investigate possible mesomorphism in such supermolecular architectures. The chemical structures have been characterized by spectral techniques and elemental analysis which establishes the identity and purity of the new compounds. The thermal behavior of the novel mesogenic oligomers has been investigated by differential scanning calorimetry and polarizing optical microscopy. These novel banana-discotic hybrid oligomers are unable to exhibit mesomorphism presumably because of the incompatibility of the bent-core with the discotic core and/or the volume fraction mismatch of the mesogenic rigid cores. Attempts to realize mesophase in such architectures with different molecular topologies are currently under way in our laboratory.

## Experimental

All the reagents and solvents were used as received without any further purification except CH_2_Cl_2_ which was dried and distilled before the reactions. Column chromatographic separation was performed on silica gel (100–200 mesh). ^1^H NMR spectra were recorded in CDCl_3_ on a 400 MHz (Bruker AMX 400) spectrometer. All chemical shifts are reported in δ (ppm) units down field from tetramethylsilane (TMS) and *J* values are reported in Hz. FT-IR spectra were recorded as KBr discs on a Shimadzu FTIR-8400 spectrophotometer. Elemental analysis was performed on a Carlo-Erba Flash 1112 analyzer. Transition temperatures were observed using a Mettler FP82HT hot stage and FP90 central processor in conjunction with an Olympus BX51 polarizing microscope. Transition temperatures and associated enthalpies were measured by differential scanning calorimetry heating from room temperature to isotropic temperatures at the scan rate of 5 °C per minute (Perkin-Elmer Model Pyris 1D).

### Synthesis

Compound **1**, compound **5** and Compound **7a–b** were prepared by following the literature procedures [28–31].

**Preparation of 2-(12-bromododecyloxy)-3,6,7,10,11-pentakis(hexyloxy)triphenylene (2):** A mixture of monohydroxy triphenylene (2.0 g, 2.7 mmol), 1,12-dibromododecane (4.4 g, 13.0 mmol), cesium carbonate (1.12 g, 8.8 mmol) and 50 ml of methyl ethyl ketone (MEK) was refluxed for 48 h at 85 °C. The resulting mixture was cooled and poured into 50 ml of water and then extracted into chloroform. The organic layer was washed with water, dried over anhydrous sodium sulfate followed by removal of solvent. The crude product was purified by column chromatography over silica gel with eluent 2% ethyl acetate in hexane. Finally the product was precipitated as waxy solid from dichloromethane by adding hexane, yield 2.44 g.

**Preparation of benzyl 4-{12-[3,6,7,10,11-pentakis(hexyloxy)triphenylene-2-yloxy]dodecyloxy}benzoate (3):** Triphenylene bromide **2** (2.34 g, 2.3 mmol), benzyl 4-hydroxy benzoate (0.45 g, 1.98 mmol) and cesium carbonate (2.0 g, 5.94 mmol) in 50 mL of MEK was refluxed for 48 h. The reaction mixture was poured into 50 mL of water and then extracted with chloroform, the chloroform layer was washed with 50 mL of water followed by drying over anhydrous sodium sulfate. The solvent was removed to yield a viscous oily residue **3** which was taken as such for hydrogenolysis.

**Preparation of 4-{12-[3,6,7,10,11-pentakis(hexyloxy)triphenylene-2-yloxy]dodecyloxy}benzoic acid (4):** A mixture of compound **3** (2.3 g) dissolved in 1,4-dioxane (20 ml) and 5% Pd-C catalyst (0.4 g) was stirred in an atmosphere of hydrogen until the required quantity of hydrogen was absorbed. The resulting mixture was filtered in hot and the solvent removed under reduced pressure. The solid material obtained was recrystallised using methanol. Yield 1.0 g. mp 68–70 °C.

**Preparation of bent-core bridged discotic dimer 6:** A mixture of compound **4** (0.5 g, 0.48 mmol), compound **5** (0.085 g, 0.24 mmol), DCC (0.22 g, 1.07 mmol) and catalytic quantity of DMAP was stirred in dry dichloromethane at room temperature for about 24 hours. The solvent was removed and the residue purified by column chromatography on silica gel using mixture of petroleum ether and ethyl acetate (2%) as eluent. Removal of solvent afforded a residue which was recrystallised from dichloromethane and hexane to afford **6**. Yield 0.2 g. IR (KBr), ν_max_ (cm^−1^): 2922, 2852, 1734, 1456, 1377, 1261, 1170, 721; ^1^H NMR (400 MHz, CDCl_3_, δ/ppm): 8.28 (d, 4H, *J* = 8.7 Hz, Ar-H), 8.16 (d, 4H, *J* = 8.0 Hz, Ar-H), 7.83 (s, 12H, Ar-H), 7.51 (t, 1H, *J* = 8.5 Hz, Ar-H), 7.36 (d, 4H, *J* = 8.7 Hz, Ar-H), 7.30 (t, 1H, *J* = 8.2 Hz, Ar-H), 7.26 (d, 2H, *J* = 8.1 Hz, Ar-H), 6.98 (d, 4H, *J* = 8.9 Hz, Ar-H), 4.24 (t, 24H, *J* = 6.4 Hz, -OCH_2_), 4.03 (t, 4H, *J* = 6.2 Hz, -OCH_2_), 1.97 (m, 28H, -CH_2_-), 1.69–1.31 (m, 92H, -CH_2_-), 0.94 (t, 30H, *J* = 6.1 Hz, -CH_3_); Elemental analysis: Found: C, 76.34; H, 9.01. C_154_H_210_O_22_ requires C, 76.64; H, 8.77.

Compounds **8a** and **8b** were prepared following the similar procedure for compound **6**. **8a**: IR (KBr), ν_max_ (cm^−1^): 2922, 2850, 1743, 1733, 1602, 1508, 1437, 1259, 1159, 1068, 721; ^1^H NMR (400 MHz, CDCl_3_, δ/ppm): 8.31 (m, 4H, Ar-H), 8.16 (d, 4H, *J* = 8.8 Hz, Ar-H), 7.83 (s, 6H, Ar-H), 7.63 (t, 1H, *J* = 3.4 Hz, Ar-H), 7.40 (m, 4H, Ar-H), 7.13–6.95 (m, 7H, Ar-H), 4.24 (t, 12H, *J* = 6.4 Hz, -OCH_2_-), 4.08 (t, 2H, *J* = 6.2 Hz, -OCH_2_-), 3.98 (t, 2H, *J* = 6.1 Hz, -OCH_2_-), 1.97–1.77 (m, 16H, -CH_2_-), 1.51–1.19 (m, 68H, -CH_2_-), 0.94 (t, 15H, *J* = 7 Hz, -CH_3_), 0.87 (t, 3H, *J* = 6.7 Hz, -CH_3_); Elemental analysis: **8a** Found: C, 75.91; H, 8.74. C_108_H_144_O_16_ requires C, 76.38; H, 8.54. **8b**: IR (KBr), ν_max_ (cm^−1^): 2922, 2852, 1726, 1606, 1462, 1377, 1263, 1169, 721; ^1^H NMR (400 MHz, CDCl_3_, δ/ppm): 8.31 (m, 3H, Ar-H), 8.15 (m, 4H, Ar-H), 7.85 (s, 6H, Ar-H), 7.61 (t, 1H, *J* = 8 Hz, Ar-H), 7.39 (m, 3H, Ar-H), 7.28 (m, 3H, Ar-H), 6.98 (m, 4H, Ar-H), 4.25 (t, 12H, *J* = 6.5 Hz, -OCH_2_-), 4.08 (m, 4H, -OCH_2_-), 2.06–1.78 (m, 16H, -CH_2_-), 1.62–1.23 (m, 68H, -CH_2_-), 0.95 (t, 15H, *J* = 6.9 Hz, -CH_3_), 0.88 (t, 3H, *J* = 6.5 Hz, -CH_3_); Elemental analysis: **8b** Found: C, 74.3; H, 8.39. C_108_H_142_O_16_F_2_ requires C, 74.79; H, 8.25.
